# Identification of crucial pathways and genes linked to endoplasmic reticulum stress in PCOS through combined bioinformatic analysis

**DOI:** 10.3389/fmolb.2024.1504015

**Published:** 2025-01-09

**Authors:** Yan Zhang, Xiujuan Chen, Yuan Lin, Xiaoqing Liu, Xiumei Xiong

**Affiliations:** Department of Obstetrics and Gynecology, Fujian Maternity and Child Health Hospital College of Clinical Medicine for Obstetrics Gynecology and Pediatrics, Fujian Medical University, Fuzhou, China

**Keywords:** PCOS, ERS, bioinformatic analysis, differentially expressed genes, hub genes

## Abstract

**Background:**

Polycystic ovary syndrome (PCOS) is a common endocrine and metabolic condition impacting millions of women worldwide. This study sought to identify granulosa cell endoplasmic reticulum stress (GCERS)-related differentially expressed genes (DEGs) between women with PCOS and those without PCOS using bioinformatics and to investigate the related molecular mechanisms.

**Methods:**

Two datasets were downloaded from GEO and analysed using the limma package to identify DEGs in two groups—PCOS and normal granulosa cells. Enrichment analyses, including GO, KEGG, and GSEA, were then conducted on the DEGs. Differential immune infiltration was assessed using CIBERSORT and correlations with immune cell biomarkers were evaluated. Networks for protein-protein interactions, transcription factor-target genes, miRNA-target genes, and drug-target genes were constructed and visualized using Cytoscape to identify key hub gene nodes. Finally, key genes were analysed for differential expression and correlated.

**Results:**

Overall, 127 co-DEGs were identified in the two datasets. Our study revealed that these DEGs were primarily associated with cell cycle arrest, p53-mediated signal transduction, drug response, and gland development, with molecular functions enriched in growth factor binding, collagen binding, and receptor protein kinase activity. GSEA revealed that the co-DEGs were primarily associated with immune and inflammatory pathways. Eleven hub genes—*MMP9*, *SPI1*, *IGF2R*, *GPBAR1*, *PDGFA*, *BMPR1A*, *LIFR*, *PRKAA1*, *MSH2*, *CDC25C*, and *KCNH2*—were identified through the PPI, TF target genes, miRNA target genes, and drug target gene networks.

**Conclusion:**

We identified several crucial genes and pathways linked to the onset and development of PCOS. Our findings offer a clear connection between PCOS and GCERS, clarify the molecular mechanisms driving PCOS progression, and offer new perspectives for discovering valuable therapeutic targets and potential biomarkers for the condition.

## 1 Background

Polycystic ovary syndrome (PCOS) is the most prevalent endocrine disorder among women of reproductive age, affecting 6%–10% of this group (Azziz et al., 2016), making it the leading cause of anovulatory infertility (Bozdag et al., 2016), impacting 26% of women globally (Deswal et al., 2020; Patel, 2018). The pathophysiology of PCOS is intricate and involves a combination of factors such as irregular gonadotropin release, excess androgen production, insulin insensitivity, and ovarian abnormalities (Dumesic et al., 2015). To date, no consensus has been arrived upon the diagnostic criteria or effective therapeutic interventions for PCOS. To date, no medication has been specifically designed to treat PCOS. Commonly used medications include antiandrogens and insulin-sensitizing agents. However, these drugs can have side effects, such as gastrointestinal problems and impaired liver and kidney functions, and require personalized medication. The long-term use of these drugs may not be an ideal solution. Understanding the pathogenesis of PCOS may contribute to improved clinical diagnosis and treatment modalities, thereby improving reproductive outcomes. Recent research suggests that intraovarian microenvironment damage is critical in promoting the development of PCOS (Xiang et al., 2023; Chiang et al., 2023; Koike et al., 2022), but the underlying mechanisms remain unclear.

Endoplasmic reticulum stress (ERS) is strongly linked to oxidative stress in the local ovarian microenvironment. Recent studies have emphasized the crucial role of granulosa cells ERS (GCERS) in ovarian microenvironment (Harada et al., 2021; Ajoolabady et al., 2023). Hyperandrogenism (HA), insulin resistance (IR), and intraovarian microenvironment damage in PCOS are closely related to ERS (Wang and Zhang, 2022). ERS is activated in PCOS granulosa cells (GCs) (Takahashi et al., 2017), and elevated androgen levels can successfully induce ERS in cultured human GCs (Azhary et al., 2019). We propose that ERS, combined with high androgen levels, oxidative stress, and inflammation, fuel a damaging cycle in the follicle of PCOS (Harada, 2022). This abnormal environment impairs granulosa cell function and promotes PCOS development. These findings indicate that targeting ERS could be a promising approach for treating PCOS. However, the extent to which these mechanisms contribute to the development of PCOS is still uncertain. Further research targeting ERS might help elucidate the pathophysiological mechanisms of PCOS and explore potential treatments for follicular developmental disorders associated with this condition.

Currently, research using bioinformatic approaches to explore the role of ERS-related genes in the progression of PCOS is limited. In this study, we analyzed two original microarray datasets from the Gene Expression Omnibus (GEO) (Clough and Barrett, 2016) database to identify differentially expressed genes (DEGs) between PCOS and GCERS and to explore the associated biological processes through comprehensive bioinformatics. Our goals were to pinpoint some key genes and pathways implicated in PCOS, identify potential novel biomarkers for diagnosis and therapy, and investigate the molecular mechanisms involved. We aimed to enhance our understanding of PCOS pathogenesis and advance the molecular insights into this condition.

## 2 Methods

### 2.1 Data collection

**FIGURE 1:**
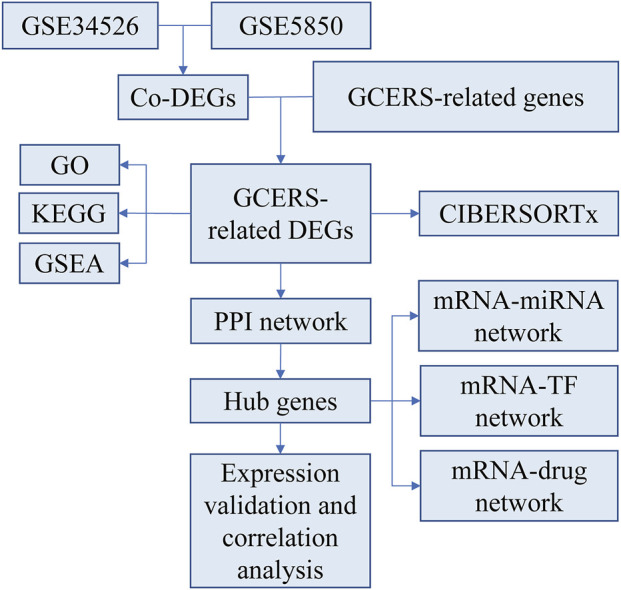
Research flow chart.

Two microarray datasets [GSE34526 (Yang et al., 2021) and GSE5850 (Wood et al., 2007)] were gathered from GEO (Barrett et al., 2013) utilizing the R package GEOquery (version 2.70.0) (Davis and Meltzer, 2007). GSE34526 included gene expression data from seven individuals with PCOS and three healthy controls, whereas GSE5850 included 12 samples from six patients with PCOS and six controls. The data were normalized using the R package limma (version 3.58.1) (Ritchie et al., 2015). GCERS-related genes were sourced from the GeneCards database (Stelzer et al., 2016) by searching for “granulosa cell endoplasmic reticulum stress,” leveraging its strengths as a comprehensive and specialized gene database. This search resulted in the identification of 1,263 genes (Supplementary Table S1). The research steps are illustrated in Figure 1.

### 2.2 Differentially expressed gene selection

We used the limma package in R to identify DEGs between PCOS and control samples. The adjusted p value and log fold changes |logFC| were calculated, with the threshold set at |logFC| > 0.5 and p < 0.05. Upregulated genes had |logFC| > 0.5, whereas downregulated genes had |logFC| < −0.5. DEGs were visualized using the “complexheatmap” and “ggplot2” R packages (version 3.4.4) to create heat maps and volcano plots. Common DEGs between PCOS- and GCERS-related genes were identified using Venn diagrams and selected for further analysis.

### 2.3 Functional classification and pathway enrichment of DEGs

Co-DEGs were analyzed for functional enrichment using the Gene Ontology (GO) (Gene Ontology, 2015) categories: biological process (BP), molecular function (MF), and cellular component (CC) and Kyoto Encyclopedia of Genes and Genomes (KEGG) (Kanehisa and Goto, 2000) pathways using the R package clusterProfiler (version 4.10.0) (Yu et al., 2012). Gene set enrichment analysis (GSEA) (Subramanian et al., 2005) was also conducted using the c2.cp.v7.2. symbol gene set from MSigDB (Liberzon et al., 2015). Enrichment results with adjusted p-values <0.05 were selected, and false discovery rate-adjusted p-values (Q values) <0.05 were used as the cutoff for GO and KEGG analyses.

### 2.4 Estimation of immune infiltration using CIBERSORT

We used the CIBERSORT (Newman et al., 2019) algorithm to estimate and compare the abundance of immune cell types between patients with PCOS and controls. Statistical significance was set at p-values <0.05. Correlations among immune cells were visualized using heat maps created using the R package ggplot2.

### 2.5 PPI network construction

We employed STRING (Szklarczyk et al., 2019) to build a protein-protein interaction (PPI) network for GCERS-related DEGs using an interaction score threshold of 0.15. PPI networks were visualized using Cytoscape (version 3.9.1) (Shannon et al., 2003), where key proteins and hub genes with high connectivity were identified. Hub gene expression levels were analyzed for differences, and correlations among these genes were assessed.

### 2.6 TF-target regulatory network construction

Target genes were predicted using CHIPBase (Zhou et al., 2017) (version 2.0) and hTFtarget (Zhang Q. et al., 2020) databases for TFs associated with GCERS-related DEGs. The resulting TF-target gene regulatory network, illustrating the connections between TFs and potential targets, was visualized using Cytoscape.

### 2.7 miRNA-target regulatory network construction

The miRNA-gene interactions for GCERS-related DEGs were predicted using the ENCORI (Starbase 3.0) (Li et al., 2014) and miRDB databases (Chen and Wang, 2020). The resulting miRNA-target gene regulatory network, which illustrated the interactions between miRNAs and their potential targets in PCOS, was visualized using Cytoscape.

### 2.8 Drug-target regulatory network construction

We used the Drug-Gene Interaction Database (DGIdb) (Freshour et al., 2021) to identify candidate drug and molecules that interact with target gene. The relationships between the drug and their potential targets were examined, and drug-target regulatory network was visualized with Cytoscape.

### 2.9 Drug sensitivity analysis

We performed drug sensitivity analysis for key genes based on the expression levels of GCERS-related DEGs and drug data from the Genomics of Drug Sensitivity in Cancer (GDSC) (Yang et al., 2013), Cancer Cell Line Encyclopedia (CCLE) (Nusinow et al., 2020), and CellMiner databases (Shankavaram et al., 2009; Reinhold et al., 2012), and presented the results.

### 2.10 Statistical analysis

Data were processed and analyzed with R software (version 4.1.2). Continuous variables are expressed as mean ± standard deviation. The Wilcoxon rank-sum test was employed for two-group comparisons, while the Kruskal–Wallis test was used for comparisons involving three or more groups. Categorical variables were assessed with the chi-square test or Fisher’s exact test. Spearman’s correlation analysis evaluated correlations between different molecules. Statistical significance was defined as p < 0.05.

## 3 Results

### 3.1 Identification of DEGs

**FIGURE 2:**
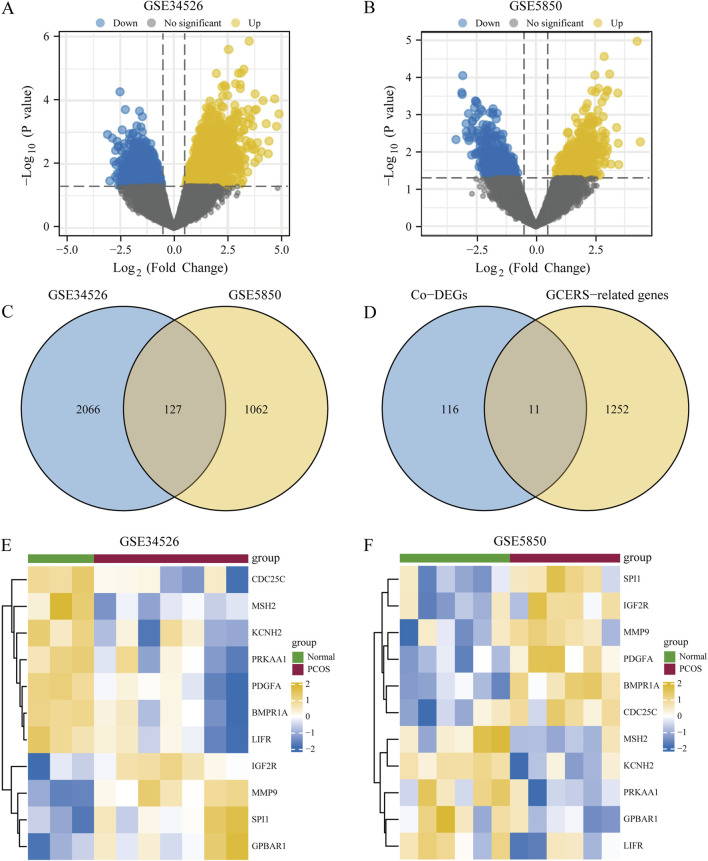
Analysis of differentially expressed genes (DEGs) associated with polycystic ovary syndrome (PCOS). **(A, B)** Volcano plots of DEG between PCOS and normal tissue in the GSE34526 dataset **(A)** and the GSE5850 dataset **(B)**. **(C)** Venn diagram of DEG in the GSE34526 and GSE5850 datasets. **(D)** Venn diagram of common differentially expressed genes (Co-DEGs) and granulosa cell endoplasmic reticulum stress-related genes within the datasets. **(E, F)** Complex numerical heat maps of granulosa cell endoplasmic reticulum stress-related differentially expressed genes in the GSE34526 **(E)** and GSE5850 **(F)** datasets. PCOS, polycystic ovary syndrome; Co-DEGs, common differentially expressed genes; GCERS, granulosa cell endoplasmic reticulum stress.

The DEGs between two groups from the GSE34526 and GSE5850 datasets are shown in Table 1. We found 2193 DEGs (1380 upregulated, 813 downregulated) in GSE34526 and 1189 DEGs (628 upregulated, 561 downregulated) in GSE5850. The DEGs of the two datasets visualised by Volcano plot analysis (Figures 2A, B), 127 overlapping DEGs were identified as common DEGs between GSE34526 and GSE5850 (Figure 2C). Venn diagram analysis identified 11 overlapping GCERS-related DEGs: *MMP9*, *SPI1*, *IGF2R*, *GPBAR1*, *PDGFA*, *BMPR1A*, *LIFR*, *PRKAA1*, *MSH2*, *CDC25C* and *KCNH2* (Table 2; Figure 2D). Heatmap analysis depicted these GCERS-related DEGs in the datasets (Figures 2E, F).

**TABLE 1:** List of PCOS dataset information.

	GSE34526	GSE5850
Platform	GPL570	GPL570
Species	*Homo sapiens*	*Homo sapiens*
Tissue	Granulosa cells	MII oocyte
Samples in PCOS group	7	6
Samples in normal group	3	6
References	PMID: 22904171	PMID: 17148555

PCOS, polycystic ovary syndrome.

**TABLE 2:** List of GCERS-related differentially expressed genes.

Gene_name	Description	log2 fold change	p value
MMP9	Matrix metallopeptidase 9	0.963633499	0.000282629
SPI1	Spi-1 proto-oncogene	2.355751449	0.00684742
IGF2R	Insulin like growth factor 2 receptor	0.356836662	0.010836028
GPBAR1	G protein-coupled bile acid receptor 1	0.850471449	0.011045476
PDGFA	Platelet derived growth factor subunit A	−2.412202784	0.012120735
BMPR1A	Bone morphogenetic protein receptor type 1A	−2.481085433	0.019431831
LIFR	LIF receptor subunit alpha	−2.026175013	0.028658683
PRKAA1	Protein kinase AMP-activated catalytic subunit alpha 1	−1.98240041	0.032168289
MSH2	MutS homolog 2	−1.533690309	0.043296104
CDC25C	Cell division cycle 25C	−1.698993042	0.043343966
KCNH2	Potassium voltage-gated channel subfamily H member 2	−1.787392585	0.047141412

GCERS, granulosa cell endoplasmic reticulum stress.

### 3.2 Enrichment analysis

**FIGURE 3:**
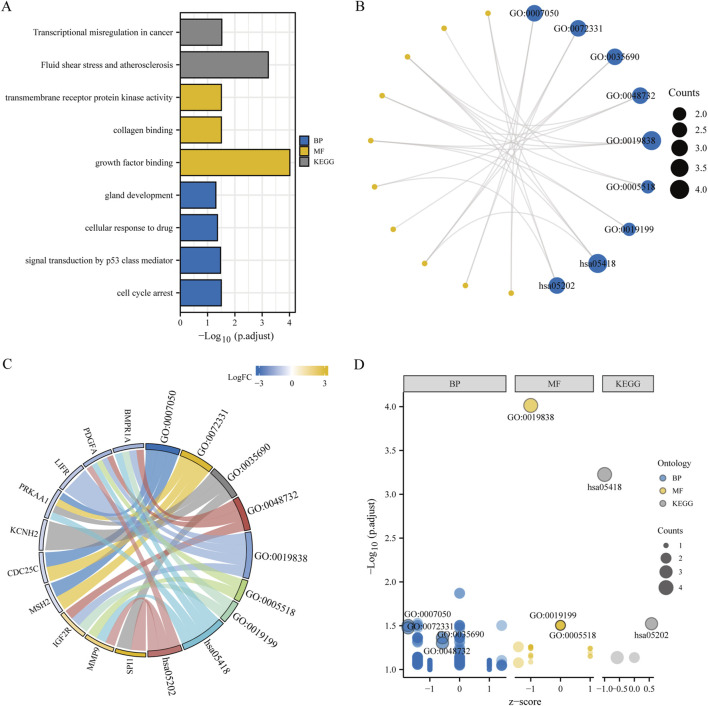
Functional enrichment analysis (GO) and pathway enrichment analysis (KEGG). **(A–D)** Bar chart **(A)**, circular network diagram **(B)**, bar graph **(C)** and bubble diagram **(D)** illustrating the results of GO and KEGG enrichment analysis for differentially expressed genes associated with granulosa cell endoplasmic reticulum stress. In the circular network diagram **(B)**, yellow dots represent specific genes and blue circles represent specific pathways. GO, Gene Ontology; BP, biological process; MF, molecular function. KEGG, Kyoto Encyclopedia of Genes and Genomes. The selection criteria for GO and KEGG enrichment items are P. adj < 0.05 and FDR value (q value) < 0.05.

**FIGURE 4:**
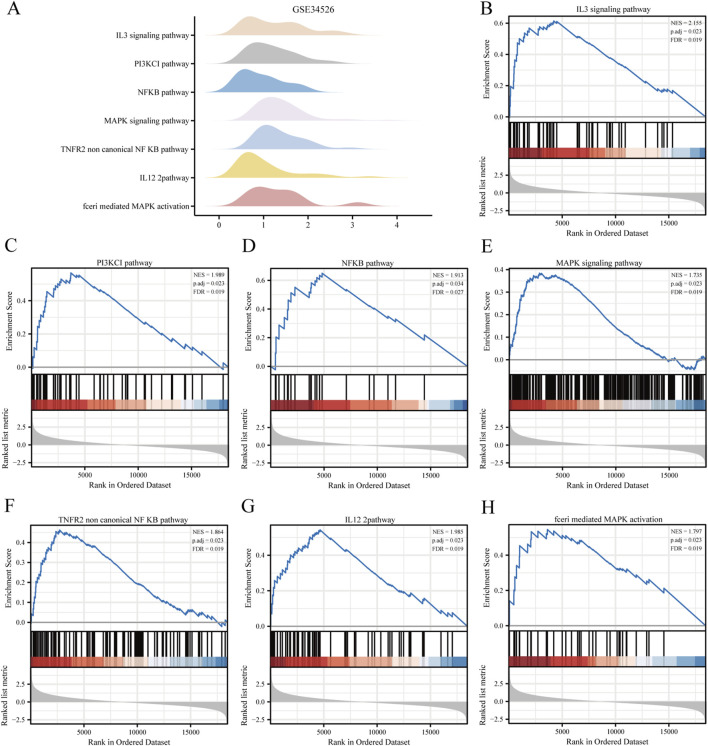
Gene set enrichment analysis (GSEA) of the PCOS dataset. **(A)** GSEA enrichment analysis of the seven most important biological traits in the GSE34526 dataset. **(B–H)** Genes in the GSE34526 dataset are significantly enriched in pathways such as IL3 signalling pathway **(B)**, PI3K-CI signalling pathway **(C)**, NFKB signalling pathway **(D)**, MAPK signalling pathway **(E)**, TNFR2 non-canonical NF-κB signalling pathway **(F)**, IL12 signalling pathway **(G)** and FcεRI-mediated MAPK activation **(H)**. The significant enrichment selection criteria for GSEA are P. adj < 0.05 and FDR value (q value) < 0.05.

Biological functions of the GCERS-related DEGs were investigated (Table 3). GO analysis highlighted enrichment in biological processes, such as cell cycle arrest, cellular response to drugs, and gland development, and in molecular functions such as growth factor/collagen binding and transmembrane receptor protein kinase activity. The results of the KEGG enrichment analysis revealed that 11 GCERS-related DEGs were significantly enriched in two KEGG pathways: “Fluid shear stress and atherosclerosis” and “Transcriptional misregulation in cancer.” The results of the GO and KEGG functional enrichment analyses are presented as a bar plot (Figure 3A). Additionally, the GO and KEGG enrichment results were visualized as a circular network diagram (Figure 3B). Next, we performed a combined logFC-based GO and KEGG enrichment analysis on these 11 GCERS-related DEGs. Based on the enrichment analysis, we calculated the z-scores for each molecule using the logFC values. The results of the combined logFC-based GO and KEGG enrichment analysis are displayed using a chord diagram (Figure 3C). Furthermore, the logFC-based GO and KEGG enrichment results are presented in a bubble chart (Figure 3D), where the majority of the enriched GO and KEGG terms are concentrated in BP pathways. GSEA further identified several pathways enriched in PCOS, including IL3, PI3K-CI, NFKB, MAPK, TNFR2 non-canonical NFKB, and IL12 signaling and FcεRI-mediated MAPK activation (Figures 4A–H; Table 4), reflecting a strong association with immune response activation and inflammation-related pathways.

**TABLE 3:** GO and KEGG enrichment analysis results of GCERS- related differentially expressed genes.

Ontology	ID	Description	Gene ratio	Bg ratio	p value	p. adjust	q value
BP	GO:0007050	Cell cycle arrest	3/11	237/18670	3.09e-04	0.031	0.017
BP	GO:0072331	Signal transduction by p53 class mediator	3/11	267/18670	4.38e-04	0.033	0.018
BP	GO:0035690	Cellular response to drug	3/11	369/18670	0.001	0.043	0.024
BP	GO:0048732	Gland development	3/11	434/18670	0.002	0.050	0.027
MF	GO:0019838	Growth factor binding	4/11	137/17697	1.09e-06	9.68e-05	5.84e-05
MF	GO:0005518	Collagen binding	2/11	67/17697	7.60e-04	0.031	0.019
MF	GO:0019199	Transmembrane receptor protein kinase activity	2/11	79/17697	0.001	0.031	0.019
KEGG	hsa05418	Fluid shear stress and atherosclerosis	4/9	139/8076	9.90e-06	5.94e-04	5.21e-04
KEGG	hsa05202	Transcriptional misregulation in cancer	3/9	192/8076	1.00e-03	0.030	0.026

GO, gene ontology; BP, biological process; MF, molecular function; KEGG, Kyoto Encyclopedia of Genes and Genomes.

**TABLE 4:** GSEA of datasets.

Description	Set size	Enrichment score	NES	p value	p. adjust
Neutrophil degranulation	450	0.599005756	2.849389421	0.001213592	0.023021781
Tyrobp causal network	59	0.749314299	2.731581114	0.001639344	0.023021781
Micrpglia pathogen phagocytosis pathway	40	0.787825725	2.669972063	0.001703578	0.023021781
Immunoregulatory interactions between a lymphoid and a non lymjoid cell	124	0.629024679	2.641831338	0.001470588	0.023021781
Leishmania infection	67	0.693773999	2.609597589	0.001592357	0.023021781
Human complement system	92	0.643016526	2.569987298	0.001519757	0.023021781
Antige processing cross presentation	95	0.625046783	2.518418196	0.001503759	0.023021781
Systemic lupus erythematosus	50	0.708603781	2.503527886	0.001675042	0.023021781
IL3 signaling pathway	49	0.61414965	2.154620678	0.001692047	0.023021781
PI3KCI pathway	48	0.567286611	1.989021484	0.001689189	0.023021781
NFKB pathway	21	0.649512946	1.913055831	0.003610108	0.033945554
MAPK signaling pathway	236	0.384271448	1.73543974	0.001308901	0.023021781
TNFR2 non canonical NF KB pathway	95	0.462539923	1.86365083	0.001503759	0.023021781
IL12 2pathway	60	0.542571064	1.985201268	0.001628664	0.023021781
Fceri mediated MAPK activation	35	0.54544542	1.79727498	0.001718213	0.023021781

GSEA, gene set enrichment analysis.

### 3.3 Immune cell infiltration analysis

**FIGURE 5:**
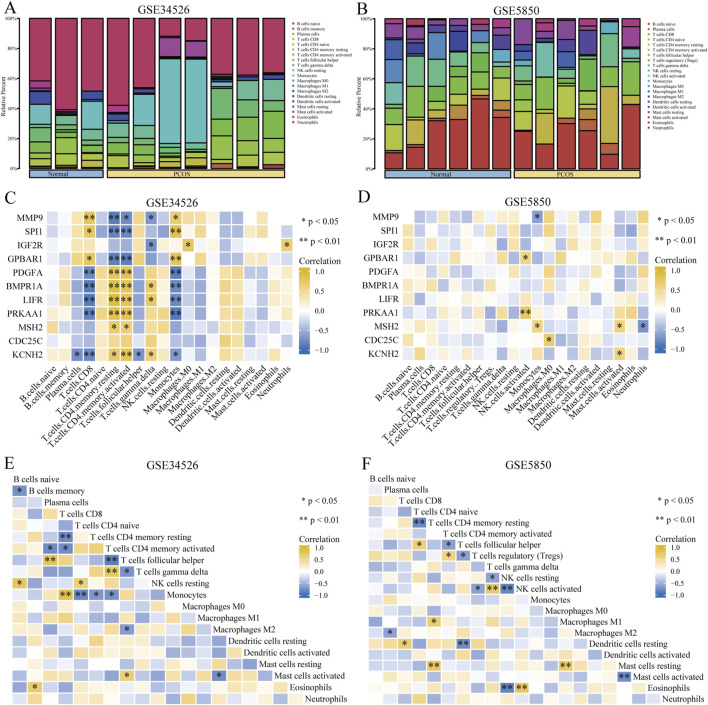
Immune infiltration analysis of the PCOS dataset. **(A, B)** Bar graph showing the infiltration results of 20 immune cell types in the GSE34526 dataset **(A)** and 21 immune cell types in the GSE5850 dataset **(B)**. **(C, D)** Heat maps showing the correlation between immune cell abundance and 11 GCERS-related DEGs in the GSE34526 **(C)** and GSE5850 **(D)** datasets. E-F. Heatmap showing the correlation between immune cells in the GSE34526 **(E)** and GSE5850 **(F)** datasets. One asterisk (*) indicates a p-value < 0.05, which is statistically significant; two asterisks (**) indicate a p-value < 0.01, which is highly statistically significant.

Our study showed that activated CD4^+^ memory T cells and T follicular helper cells were predominant in both the PCOS and control groups (Figures 5A, B). The association between eleven GCERS-related DEGs and infiltrating immune cells was analysed (Figures 5C, D). Correlation analysis in dataset GSE34526 showed that activated CD4^+^ memory T cells was linked with nine GCERS-related DEGs (*MMP9*, *SPI1*, *GPBAR1*, *PDGFA*, *BMPR1A*, *LIFR*, *PRKAA1*, *MSH2*, and *KCNH2*) with consistent correlation directions (p < 0.05). Additionally, T cells, CD8 cells, and monocytes were associated with eight DEGs (*MMP9*, *SPI1*, *GPBAR1*, *PDGFA*, *BMPR1A*, *LIFR*, *PRKAA*, and *KCNH2*). T follicular helper cells correlated with five DEGs (*MMP9*, *IGF2R, BMPR1A*, *LIFR*, and *KCNH2*). Dataset GSE5850 highlighted significant correlations between activated NK cells and BMPR1A/PRKAA1, monocytes and MMP9/MSH2, activated mast cells and MSH2/KCNH2, M0 Macrophages and CDC25C, and neutrophils and MSH2. A correlation heatmap illustrated significant correlation between activated CD4^+^ memory T cells and T follicular helper cells across the datasets (Figures 5E, F).

### 3.4 Integrated PPI network construction

**FIGURE 6:**
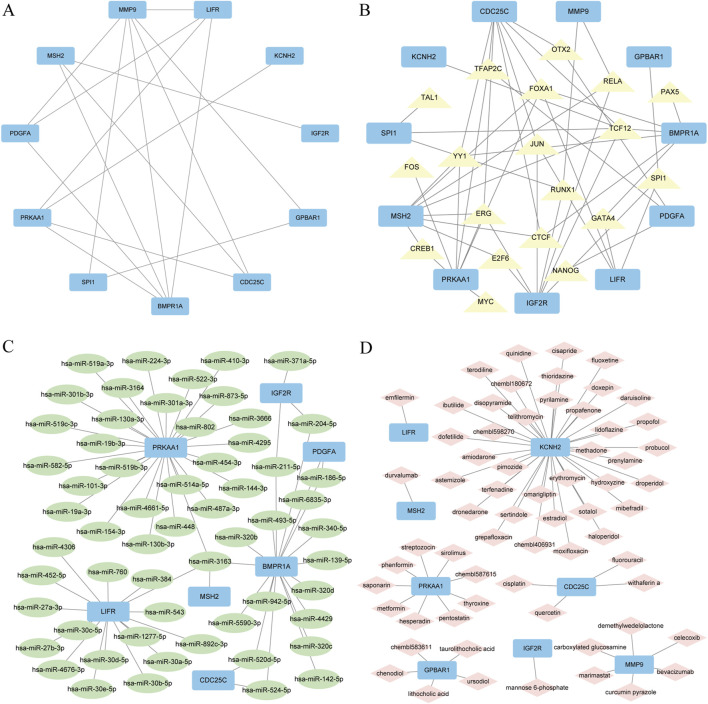
Regulatory networks of PPI, mRNA-TF, mRNA-miRNA, and mRNA-drug. **(A)** PPI regulatory network for ER stress-related differentially expressed genes in granulosa cells. **(B)** mRNA-TF regulatory network, where blue rectangles represent mRNAs and yellow triangles represent transcription factors (TFs). **(C)** mRNA-miRNA regulatory network, where blue rectangles represent mRNAs and green ellipses represent miRNAs. **(D)** mRNA-drug regulatory network, where blue rectangles represent mRNAs and pink diamonds represent drugs. PPI, protein-protein interaction; TF, transcription factor.

Using the STRING database (with a minimum interaction score set to 0.150), we constructed a protein-protein interaction (PPI) network for 11 differentially expressed genes (DEGs) associated with GCERS. These genes include MMP9, SPI1, IGF2R, GPBAR1, PDGFA, BMPR1A, LIFR, PRKAA1, MSH2, CDC25C, and KCNH2. The network was visualized using Cytoscape software, revealing multiple interactions between the genes (Figure 6A). Specifically, MMP9 is connected to other genes through six edges, and BMPR1A through five edges. These dense interactions suggest that they may play a crucial role in the core regulatory network of GCERS.

### 3.5 Integrated TF-target network construction

By integrating the TF target gene results with the TF interaction network, 11 GCERS-related DEGs and 19 TFs were identified (Figure 6B). The results revealed: 6 TFs modulated *BMPR1A*, 8 TFs controlled *CDC25C*, 1 TF affected *GPBAR1*, 7 TFs influenced *IGF2R*, 1 TF targeted *KCNH2*, 4 TFs modulated *LIFR*, 2 TFs regulated *MMP9*, 8 TFs affected *MSH2*, 3 TFs affected *PDGFA*, 7 TFs regulated *PRKAA1*, and 3 TFs controlled *SPI1* (Supplementary Table S2).

### 3.6 Integrated miRNA-target network construction

By integrating the results of the miRNA target genes with the miRNA interaction network, we identified seven GCERS-related DEGs and 59 miRNAs (Figure 6C).The results revealed: 17 miRNAs (e.g., miR-186-5p) controlled *BMPR1A*, 2 miRNAs (e.g., miR-524-5p) affected *CDC25C*, 3 miRNAs (e.g., miR-204-5p) influenced *IGF2R*, 16 miRNAs (e.g., miR-543) targeted *LIFR*, 1 miRNA (miR-3163) regulated *MSH2*, 2 miRNAs (e.g., miR-6835-3p) controlled *PDGFA*, and 27 miRNAs (e.g., miR-3163) targeted *PRKAA1* (Supplementary Table S3).

### 3.7 Integrated drug-target network construction

After combining the drug-target gene results with the drug–interactive network, 8 GCERS-related DEGs and 64 drugs were identified. The genes and drugs used are shown in Figure 6D. The results revealed: 6 drugs (e.g., celecoxib) regulated *MMP9*, 1 drug (mannose-6-phosphate) regulated *IGF2R*, 5 drugs (e.g., taurolithocholic acid) regulated *IGF2R*, 1 drug (emfilermin) regulated *LIFR*, 1 drug (durvalumab) regulated *MSH2*, 9 drugs (e.g., metformin) regulated *PRKAA1*, 4 drugs (e.g., fluorouracil) regulated *CDC25C*, and 37 drugs (e.g., estradiol) regulated *KCNH2* (Supplementary Table S4).

### 3.8 Correlation analysis of key genes

**FIGURE 7:**
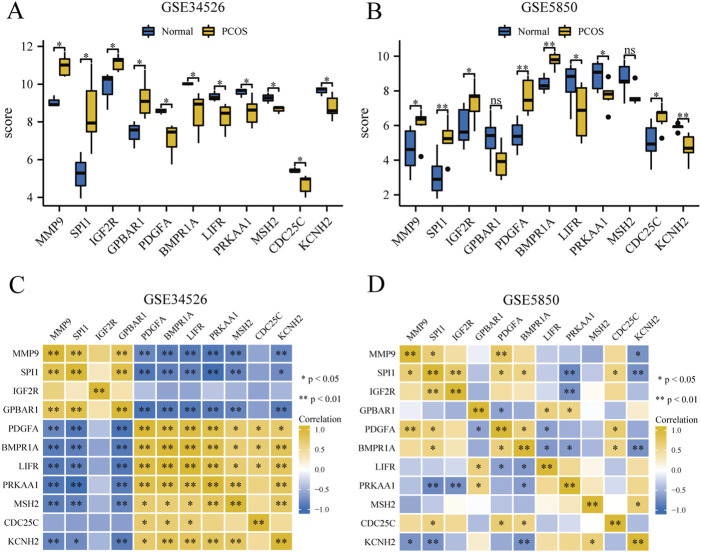
Expression and correlation analysis of hub genes in the PCOS dataset. **(A, B)** Differential expression analysis of hub genes in the GSE34526 dataset **(A)** and the GSE5850 dataset **(B)**. **(C, D)** Correlation analysis between hub genes in GSE34526 dataset **(C)** and GSE5850 dataset **(D)**. “ns” indicates a p-value ≥ 0.05, indicating no statistically significant difference; one asterisk (*) indicates a p-value < 0.05, indicating a statistically significant difference; two asterisks (**) indicate a p-value < 0.01, indicating a highly statistically significant difference.

We compared 11 GCERS-related DEGs between two groups in datasets GSE34526 and GSE5850 (Figures 7A, B). In GSE34526 cells (Figure 7A), all hub genes showed statistically significant differences. In GSE5850 (Figure 7B), nine hub genes showed significant differences, excluding *GPBAR1* and *MSH2*.

To explore the correlations between the hub genes, we performed a correlation analysis for the 11 hub genes (Figures 7C, D). In the GSE34526 dataset, *CDC25C* gene had statistically significant correlations with three hub genes (*PDGFA*, *BMPR1A*, and *LIFR*), and nine hub genes (*MMP9, SPI1, GPBAR1, PDGFA, BMPR1A, LIFR, PRKAA1, MSH2,* and *KCNH2*) exhibited statistically significant correlations among themselves. In the GSE5850 dataset, *SPI1* had the most significant correlation with seven hub genes (*MMP9, SPI1, IGF2R, PDGFA, BMPR1A, PRKAA1, CDC25C,* and *KCNH2*), whereas *MSH2* had the least significant correlation, with only one significant correlation with *KCNH2*.

### 3.9 Drug sensitivity analysis

**FIGURE 8:**
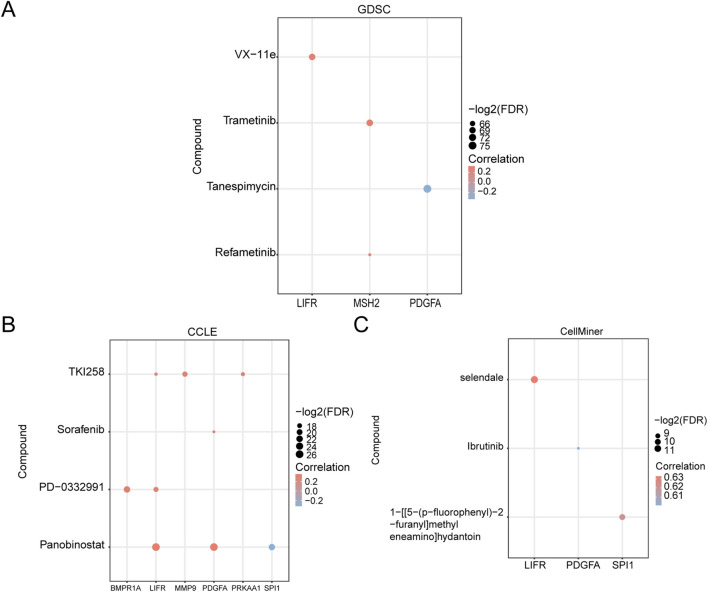
Drug Sensitivity Analysis. **(A–C)** Drug sensitivity analysis based on GCERS-related DEGs using the Genomics of Drug Sensitivity in Cancer (GDSC) database **(A)**, Cancer Cell Line Encyclopedia (CCLE) database **(B)**, and CellMiner database **(C)**. GDSC, Genomics of Drug Sensitivity in Cancer; CCLE, Cancer Cell Line Encyclopedia. The absolute value of the correlation coefficient (r) is classified as weak or no correlation if <0.3, weak correlation if between 0.3 and 0.5, moderate correlation if between 0.5 and 0.8, and strong correlation if >0.8. Red represents positive correlation, and blue represents negative correlation.

We analyzed the drug sensitivity of GCERS-related DEGs using mRNA expression profiles and drug activity data from three different databases: GDSC, CCLE, and CellMiner. The pRRophetic algorithm was employed to predict the sensitivity of GCERS-related DEGs to common anticancer drugs based on their expression levels. A ridge regression model was constructed, and drug sensitivity was estimated using IC50 values. Additionally, the correlation between GCERS-related DEGs and drug molecules was visualized across the three databases. The results indicated that in the GDSC database, four drugs with significant interactions with GCERS-related DEGs were identified (Figure 8A): VX-11e, Trametinib, Tanespimycin, and Refametinib. In the CCLE database, four drugs with similar interactions were found (Figure 8B): TKI258, Sorafenib, PD-0332991, and Panobinostat. In the CellMiner database, three drugs with interactive relationships were identified (Figure 8C): Selendale, Ibrutinib, and 1-[[5-(p-fluorophenyl)-2-furanyl]methyleneamino]hydantoin.

## 4 Discussion

PCOS is characterized by elevated levels of androgens, irregular menstrual cycles, as well as dysfunction of the ovaries (Shi et al., 2020). GCs are crucial for oocyte development, including growth, meiosis, and differentiation before ovulation (Zhang et al., 2018). Recent studies have linked PCOS to several factors, particularly those related to chronic inflammation and oxidative stress (Cozzolino and Seli, 2020), which can activate ERS, leading to the apoptosis of GCs and disruption of follicular development (Lima et al., 2018). However, the clinical manifestations of PCOS are highly variable, leading to a lack of consensus regarding its pathogenesis. Thus, we aimed to identify specific and sensitive biomarkers to clarify ERS mechanisms in GCs and enhance our understanding of PCOS pathogenesis.

ER stress is a cellular adaptive mechanism that is crucial for maintaining cellular homeostasis and cellular stress. Intense or prolonged stress can shift the unfolded protein response from a protective to harmful state, culminating in the initiation of apoptotic pathways. Recent studies highlights ERS’s role in PCOS, showing that ERS contributes to the development of the condition and that ERS inhibitors can improve various aspects of PCOS (Xiang et al., 2023; Harada et al., 2021; Koike et al., 2023). This indicates that targeting ERS may be a promising treatment strategy for PCOS. However, the integrated regulatory processes involving GCs and ERS in PCOS remain unclear. Understanding the molecular mechanisms underlying PCOS is vital for advancing its diagnosis and treatment. Despite the identification of several PCOS-related biomarkers as potential therapeutic targets, the complex gene regulatory mechanisms involved in PCOS progression are not fully understood. Thus, research into these mechanisms and early interventions may have significant clinical benefits.

We identified 11 key genes related to GCERS in the PPI network, which suggests that these genes may be crucial in PCOS. *MMP9*, a zinc-dependent metalloproteinase involved in the degradation of extracellular matrix proteins, is implicated in various physiological and pathological processes, including cell signaling, immune response modulation, and tumor metastasis (Pang et al., 2024; Lu et al., 2022). *MMP9* contributes to the development of chronic inflammatory and autoimmune diseases (Bai et al., 2024; Chen et al., 2022; Amjadi et al., 2022), and plays a crucial role in hormone regulation and the maintenance of ovarian function. Consistent with our findings, *MMP9* is overexpressed in PCOS, closely associated with ovarian cyst formation and follicular atrophy (Dambala et al., 2019), and its expression can be modulated by pharmacological treatment (Shamsi et al., 2023; Yu et al., 2022), underscoring its significant role in the pathophysiology of PCOS. During ER stress, multiple transcription factors regulate *MMP9* expression. The pro-inflammatory cytokine tumor necrosis factor alpha (*TNFα*) activates the ER stress response via c-Fos, leading to *MMP9* induction. TNFα stimulation increases ER stress markers (*BiP/GRP78, XBP1, GRP94*), while inhibiting c-Fos signaling with TUDCA or 4PBA suppresses TNFα-induced *MMP9* expression and secretion (Choi et al., 2019; Woodward et al., 2020). The m6A-binding protein YTHDF1 also plays a key role in regulating both ER stress and *MMP9* (He et al., 2022). Oxidized low-density lipoprotein (oxLDL) could alter ER function, induce ER stress, and activate *MMP9* in macrophages (Sanda et al., 2017). Additionally, ER stress pathways, including *PERK, IRE1α*, and *ATF6*, are closely linked to *MMP9* expression (Chen et al., 2020; Nan et al., 2022; Maddineni et al., 2021). These findings provide new insights into the regulatory mechanisms connecting ER stress and *MMP9*, indicating that the activity of *MMP9* might influence the ER stress-induced cell death pathway (Walter et al., 2020).

*PRKAA1* encodes the AMPK protein, which plays a central role in regulating glycolysis, fatty acid oxidation, and cell proliferation and apoptosis (Zhang Y. et al., 2020; Yong et al., 2024). The metabolic regulatory mechanisms of *PRKAA1* vary across different cell types; for instance, it regulates lipid synthesis in adipocytes and glucose metabolism in hepatocytes (Yang et al., 2022). PRKAA1 influences female fertility, endometrial regeneration, and hormone regulation (Kurowska et al., 2020), potentially contributing to PCOS development by affecting insulin sensitivity (Tao et al., 2019), thereby playing a crucial role in reproductive health and metabolic balance in the condition (McCallum et al., 2018). Our research showed that *PRKAA1* is downregulated in PCOS, which is consistent with a previous study that found impaired *PRKAA1* activation in patients with PCOS (Randriamboavonjy et al., 2015). *PRKAA1* activity is closely associated with ER stress-related signaling pathways, including mTORC1, and its activation upregulates stress markers such as HSPA5, protecting cells from ER stress-induced damage (Xie et al., 2023; Yang et al., 2020; Zhou et al., 2022). Dysregulated *PRKAA1* expression is linked to several ER stress-related diseases, including metabolic disorders (e.g., diabetes, cardiovascular diseases) and tumor invasiveness (Yong et al., 2024; Zhang et al., 2022; Shao et al., 2024). By modulating the ER stress response, *PRKAA1* influences cellular metabolism, survival, and disease progression (Tan et al., 2023), making it a critical regulator and potential therapeutic target. Further research is needed to investigate the relationship between *PRKAA1* and ER stress in PCOS and explore its therapeutic potential in PCOS treatment.

*GPBAR1* is a transmembrane receptor that is widely expressed in a variety of tissues and plays a critical role in regulating numerous physiological processes, including metabolism, immune responses, and inflammation (Biagioli et al., 2023a; Chen et al., 2023; Marchiano et al., 2022). The activation of *GPBAR1* has been shown to significantly reduce the expression of markers associated with ER stress, while alleviating cell apoptosis, oxidative stress, and improving cell survival (Dicks et al., 2021; Biagioli et al., 2024). Furthermore, *GPBAR1* activation enhances the autophagic process, facilitates the clearance of unfolded proteins, and notably reduces the levels of pro-inflammatory cytokines, such as IL-6 and TNF-α, thereby inhibiting the inflammatory response triggered by ER stress (Biagioli et al., 2023b; Carino et al., 2021). Our findings align with previous studies that have demonstrated a significant downregulation of *GPBAR1* expression in PCOS (Huffman et al., 2021). This reduction in *GPBAR1* expression appears to be closely linked to ovarian dysfunction and hormone imbalance observed in PCOS patients (Li et al., 2021). We hypothesize that decreased *GPBAR1* expression in PCOS ovaries disrupts autophagic pathways and cell survival, leading to increased ER stress and impaired ovarian function. Further research is needed to elucidate the signaling pathways linking *GPBAR1* to ER stress and its role in PCOS pathophysiology, which could uncover novel therapeutic targets for this common condition.

LIFR, a key member of the IL-6 cytokine family, plays a crucial role in various biological processes, including cell proliferation, differentiation, and survival, as well as in the regulation of inflammation and immune responses (Zhao et al., 2024; Daghestani et al., 2022). Upon binding its ligand LIF, LIFR forms a heterodimeric complex that activates downstream signaling pathways (e.g., JAK/STAT, PI3K/AKT, MAPK), modulating macrophage phenotype and inflammatory responses to indirectly regulate ER stress (Viswanadhapalli et al., 2022; Feng et al., 2023). This promotes cell survival and tissue repair, highlighting the protective role of LIFR under ER stress. Reduced LIFR expression, conversely, contributes to increased apoptosis and tissue dysfunction (Huo et al., 2019; Ding et al., 2021). Consistent with previous studies (Zhao et al., 2024; Dhadhal and Nampoothiri, 2022; Javidan et al., 2022), our research shows a downregulation of LIFR in tissues from patients with polycystic ovary syndrome (PCOS), suggesting that LIFR may influence ovarian function and embryo development by modulating the ER stress response. IGF2R, an important cell membrane receptor with tissue-specific expression, is particularly prominent in the reproductive system (Guo et al., 2023). By enhancing cellular sensitivity to insulin-like growth factors (IGFs) (Wang et al., 2020), IGF2R promotes cell survival and recovery, mitigating endoplasmic reticulum stress and improving embryonic development (Ponsford et al., 2021; Murillo-Rios et al., 2017). In line with previous studies (Kaur et al., 2012; Haouzi et al., 2012), our results show IGF2R overexpression in PCOS, which contributes to metabolic dysfunction and follicular arrest, reducing oocyte developmental competence (Stubbs et al., 2013). Additionally, IGF2R genetic polymorphisms may influence PCOS pathogenesis, suggesting its potential as a biomarker for clinical diagnosis and treatment.

The etiology of PCOS is complex and multifactorial. It is characterized by a state of chronic inflammation (Patel, 2018; Yin et al., 2019), which leads to the activation of immune cells that infiltrate the local environment, resulting in dysregulation of the immune system (Hu et al., 2020). Beyond reproductive dysfunction, the pathogenic mechanisms involve the interplay between the immune system and reproductive processes, leading to a spectrum of alterations in cytokine profiles and immune cell dynamics. Our study found that T cells play a important role in PCOS pathophysiology, with results aligning with previous research (Chen et al., 2022; Liu et al., 2023). We observed increased infiltration of activated CD4^+^ memory T cells and T follicular helper cells, which secrete inflammatory and immunomodulatory molecules that affect ovarian function in patients with PCOS. Additionally, T cell subpopulations in granulosa cells are dysregulated because of hormonal imbalances. Our findings also showed that 11 GCERS-related DEGs were closely associated with immune cell infiltration. However, further research is needed to clarify these complex relationships.

In this study, we observed significant discrepancies in the correlation between GCERS-related genes and immune infiltrating cells across the two datasets. These differences can be attributed to several factors. First, variations in sample characteristics, such as patient age, disease duration, and other clinical parameters, could influence immune cell composition. Additionally, differences in experimental design and data processing methods may reduce dataset comparability. Moreover, the complex and multifactorial nature of polycystic ovary syndrome (PCOS), driven by genetic, environmental, and hormonal factors, leads to individual variations in immune status, further contributing to the observed heterogeneity. Despite these challenges, integrating multiple datasets remains valuable, as it allows for the identification of common patterns and enhances our understanding of the relationship between GCERS-related genes and immune infiltration in PCOS, potentially guiding future clinical interventions.

## 5 Limitations

In this study, our bioinformatic analysis identified 11 key genes and associated regulatory networks potentially involved in the pathogenesis of polycystic ovary syndrome (PCOS), suggesting promising diagnostic and therapeutic targets. However, several limitations may affect the validity and generalizability of our findings. First, the two datasets used (GSE34526 and GSE5850) had relatively small sample sizes, which may not fully represent the diversity of the PCOS population, particularly regarding age and racial differences. Moreover, we focused exclusively on the top 11 hub genes, and the precise molecular mechanisms underlying their role in PCOS remain unclear.

Second, although differential expression analysis was performed using the R package limma, these analyses rely on existing biological databases, which may limit the exploration of certain key genes’ functions. Additionally, due to technical and resource constraints, we could not conduct experimental validation, which could influence our understanding of the functional roles of the 11 genes associated with granulosa cell endoplasmic reticulum stress (GCERS). To validate these findings and better understand the molecular mechanisms of these hub genes in PCOS, further basic and clinical studies are needed.

Furthermore, as this is a cross-sectional study, it cannot establish causal relationships between gene expression and PCOS progression. Our results are primarily based on correlational analysis. The immune cell infiltration analysis relied on the CIBERSORTx model, which may have limited predictive capacity. Additionally, the two datasets used were sourced from oocytes and granulosa cells, respectively, which could introduce variations due to differing sample origins, potentially affecting gene expression interpretations. To mitigate this, we conducted separate analyses for each dataset and identified the intersection of DEGs to ensure the reliability of our results. Future research will focus on using datasets from consistent sample sources to enable more in-depth analysis, thereby enhancing the robustness and scientific validity of our conclusions. Larger-scale experimental validation and longitudinal studies are also needed to further explore the underlying mechanisms.

## 6 Conclusion

In conclusion, our bioinformatic analysis of GEO database samples identified key genes and pathways associated with PCOS. Eleven significant hub genes (*MMP9, SPI1, IGF2R, GPBAR1, PDGFA, BMPR1A, LIFR, PRKAA1, MSH2, CDC25C,* and *KCNH2*) likely play crucial roles in PCOS pathophysiology. We also predicted potential TFs, miRNAs, and drugs that might be involved in PCOS via immune and inflammatory responses. Our results may help to develop early diagnostic strategies and identify prognostic biomarkers and treatment targets. Additional studies are needed to verify these interactions and their functions.

## Data Availability

The original contributions presented in the study are included in the article/Supplementary Material, further inquiries can be directed to the corresponding author.

## Supplementary material

SUPPLEMENTARY TABLE S1 List of GCERS-related genes.

SUPPLEMENTARY TABLE S2 TF list of mRNA-TF network.

SUPPLEMENTARY TABLE S3 miRNA list of mRNA-miRNA network.

SUPPLEMENTARY TABLE S4 Drug list of mRNA-drug network.
