# Supplementary material for: Identification of crucial pathways and genes linked to endoplasmic reticulum stress in PCOS through combined bioinformatic analysis
Source: Front Mol Biosci. 2025 Jan 9;11:1504015. doi: 10.3389/fmolb.2024.1504015 (PMC11754070; doi:10.3389/fmolb.2024.1504015)
Supplement: Supplementary file 1 [file Table2.docx]

Table S2. TF list of mRNA-TF network

| gene name | TF |
| --- | --- |
| CDC25C | FOXA1 |
| MSH2 | FOXA1 |
| PRKAA1 | FOXA1 |
| MSH2 | CTCF |
| LIFR | FOXA1 |
| BMPR1A | FOXA1 |
| BMPR1A | CTCF |
| CDC25C | CTCF |
| MMP9 | RELA |
| LIFR | GATA4 |
| CDC25C | TFAP2C |
| IGF2R | RUNX1 |
| PDGFA | NANOG |
| BMPR1A | GATA4 |
| PDGFA | TFAP2C |
| CDC25C | JUN |
| MMP9 | RUNX1 |
| SPI1 | RUNX1 |
| CDC25C | OTX2 |
| IGF2R | ERG |
| IGF2R | JUN |
| MSH2 | CREB1 |
| PRKAA1 | CREB1 |
| BMPR1A | PAX5 |
| CDC25C | TCF12 |
| IGF2R | RELA |
| KCNH2 | TCF12 |
| MSH2 | TCF12 |
| MSH2 | RELA |
| MSH2 | YY1 |
| PRKAA1 | ERG |
| PRKAA1 | TFAP2C |
| PRKAA1 | YY1 |
| SPI1 | TCF12 |
| BMPR1A | YY1 |
| BMPR1A | TCF12 |
| CDC25C | ERG |
| CDC25C | YY1 |
| GPBAR1 | SPI1 |
| IGF2R | SPI1 |
| IGF2R | E2F6 |
| IGF2R | TCF12 |
| LIFR | SPI1 |
| LIFR | JUN |
| MSH2 | E2F6 |
| MSH2 | ERG |
| PDGFA | OTX2 |
| PRKAA1 | FOS |
| PRKAA1 | MYC |
| SPI1 | TAL1 |

TF，Transcription factor
