# Supplementary material for: Identification of crucial pathways and genes linked to endoplasmic reticulum stress in PCOS through combined bioinformatic analysis
Source: Front Mol Biosci. 2025 Jan 9;11:1504015. doi: 10.3389/fmolb.2024.1504015 (PMC11754070; doi:10.3389/fmolb.2024.1504015)
Supplement: Supplementary file 2 [file Table3.docx]

Table S3. miRNA list of mRNA-miRNA network

| gene name | miRNA |
| --- | --- |
| BMPR1A | hsa-miR-186-5p |
| BMPR1A | hsa-miR-6835-3p |
| LIFR | hsa-miR-543 |
| PRKAA1 | hsa-miR-3163 |
| LIFR | hsa-miR-3163 |
| MSH2 | hsa-miR-3163 |
| PDGFA | hsa-miR-6835-3p |
| LIFR | hsa-miR-27a-3p |
| LIFR | hsa-miR-27b-3p |
| BMPR1A | hsa-miR-204-5p |
| BMPR1A | hsa-miR-211-5p |
| PRKAA1 | hsa-miR-101-3p |
| PRKAA1 | hsa-miR-154-3p |
| PRKAA1 | hsa-miR-487a-3p |
| PRKAA1 | hsa-miR-519c-3p |
| PRKAA1 | hsa-miR-519b-3p |
| PRKAA1 | hsa-miR-522-3p |
| PRKAA1 | hsa-miR-519a-3p |
| PRKAA1 | hsa-miR-224-3p |
| LIFR | hsa-miR-1277-5p |
| BMPR1A | hsa-miR-139-5p |
| BMPR1A | hsa-miR-3163 |
| PRKAA1 | hsa-miR-873-5p |
| CDC25C | hsa-miR-524-5p |
| CDC25C | hsa-miR-520d-5p |
| LIFR | hsa-miR-760 |
| PDGFA | hsa-miR-186-5p |
| PRKAA1 | hsa-miR-410-3p |
| PRKAA1 | hsa-miR-582-5p |
| PRKAA1 | hsa-miR-3164 |
| BMPR1A | hsa-miR-942-5p |
| LIFR | hsa-miR-384 |
| PRKAA1 | hsa-miR-448 |
| PRKAA1 | hsa-miR-514a-5p |
| BMPR1A | hsa-miR-524-5p |
| BMPR1A | hsa-miR-520d-5p |
| PRKAA1 | hsa-miR-144-3p |
| PRKAA1 | hsa-miR-4661-5p |
| BMPR1A | hsa-miR-142-5p |
| BMPR1A | hsa-miR-493-5p |
| BMPR1A | hsa-miR-340-5p |
| BMPR1A | hsa-miR-5590-3p |
| LIFR | hsa-miR-30a-5p |
| LIFR | hsa-miR-30c-5p |
| LIFR | hsa-miR-30d-5p |
| LIFR | hsa-miR-30b-5p |
| LIFR | hsa-miR-30e-5p |
| LIFR | hsa-miR-4306 |
| PRKAA1 | hsa-miR-19a-3p |
| PRKAA1 | hsa-miR-19b-3p |
| IGF2R | hsa-miR-204-5p |
| IGF2R | hsa-miR-211-5p |
| LIFR | hsa-miR-452-5p |
| LIFR | hsa-miR-4676-3p |
| LIFR | hsa-miR-892c-3p |
| PRKAA1 | hsa-miR-802 |
| BMPR1A | hsa-miR-320b |
| BMPR1A | hsa-miR-320c |
| BMPR1A | hsa-miR-320d |
| BMPR1A | hsa-miR-4429 |
| IGF2R | hsa-miR-371a-5p |
| PRKAA1 | hsa-miR-130a-3p |
| PRKAA1 | hsa-miR-301a-3p |
| PRKAA1 | hsa-miR-130b-3p |
| PRKAA1 | hsa-miR-454-3p |
| PRKAA1 | hsa-miR-301b-3p |
| PRKAA1 | hsa-miR-4295 |
| PRKAA1 | hsa-miR-3666 |
