# Supplementary material for: Identification of crucial pathways and genes linked to endoplasmic reticulum stress in PCOS through combined bioinformatic analysis
Source: Front Mol Biosci. 2025 Jan 9;11:1504015. doi: 10.3389/fmolb.2024.1504015 (PMC11754070; doi:10.3389/fmolb.2024.1504015)
Supplement: Supplementary file 4 [file Table4.docx]

Table S4. Drug list of mRNA-drug network

| gene name | drug |
| --- | --- |
| MMP9 | marimastat |
| MMP9 | curcumin pyrazole |
| MMP9 | bevacizumab |
| MMP9 | carboxylated glucosamine |
| MMP9 | demethylwedelolactone |
| MMP9 | celecoxib |
| IGF2R | mannose 6-phosphate |
| GPBAR1 | ursodiol |
| GPBAR1 | chembl583611 |
| GPBAR1 | taurolithocholic acid |
| GPBAR1 | chenodiol |
| GPBAR1 | lithocholic acid |
| LIFR | emfilermin |
| PRKAA1 | phenformin |
| PRKAA1 | hesperadin |
| PRKAA1 | sirolimus |
| PRKAA1 | streptozocin |
| PRKAA1 | metformin |
| PRKAA1 | thyroxine |
| PRKAA1 | pentostatin |
| PRKAA1 | saponarin |
| PRKAA1 | chembl587615 |
| MSH2 | durvalumab |
| CDC25C | withaferin a |
| CDC25C | cisplatin |
| CDC25C | quercetin |
| CDC25C | fluorouracil |
| KCNH2 | astemizole |
| KCNH2 | sertindole |
| KCNH2 | disopyramide |
| KCNH2 | propafenone |
| KCNH2 | thioridazine |
| KCNH2 | erythromycin |
| KCNH2 | dofetilide |
| KCNH2 | terfenadine |
| KCNH2 | sotalol |
| KCNH2 | cisapride |
| KCNH2 | quinidine |
| KCNH2 | ibutilide |
| KCNH2 | amiodarone |
| KCNH2 | pimozide |
| KCNH2 | fluoxetine |
| KCNH2 | dronedarone |
| KCNH2 | doxepin |
| KCNH2 | hydroxyzine |
| KCNH2 | lidoflazine |
| KCNH2 | omarigliptin |
| KCNH2 | telithromycin |
| KCNH2 | pyrilamine |
| KCNH2 | grepafloxacin |
| KCNH2 | propofol |
| KCNH2 | chembl598270 |
| KCNH2 | chembl180672 |
| KCNH2 | chembl406931 |
| KCNH2 | methadone |
| KCNH2 | estradiol |
| KCNH2 | droperidol |
| KCNH2 | probucol |
| KCNH2 | terodiline |
| KCNH2 | moxifloxacin |
| KCNH2 | prenylamine |
| KCNH2 | haloperidol |
| KCNH2 | mibefradil |
| KCNH2 | daruisoline |
